# A first in man, dose-finding study of the mTORC1/mTORC2 inhibitor OSI-027 in patients with advanced solid malignancies

**DOI:** 10.1038/bjc.2016.59

**Published:** 2016-03-22

**Authors:** Joaquin Mateo, David Olmos, Herlinde Dumez, Srinivasu Poondru, Nancy L Samberg, Sharon Barr, Jan M Van Tornout, Fei Jie, Shahneen Sandhu, Daniel S Tan, Victor Moreno, Patricia M LoRusso, Stan B Kaye, Patrick Schöffski

**Affiliations:** 1 Drug Development Unit; The Royal Marsden NHS Foundation Trust and The Institute of Cancer Research, London SM2 5PT, UK; 2 Prostate Cancer Clinical Research Unit, Spanish National Cancer Research Centre (CNIO), Madrid 28029, Spain; 3 Department of Oncology, University Hospitals Leuven and KU Leuven, Leuven B-3000, Belgium; 4 Astellas Pharma Global Development, Northbrook, IL 60201, USA; 5 Yale University, New Haven, CT 06520, USA

**Keywords:** mTORC1/2, first in man, phase I clinical trial, pharmacodynamics, pharmacokinetics

## Abstract

**Background:**

The kinase activity of mTOR involves 2 multiprotein complexes, (mTORC1-mTORC2). Targeting mTORC1 with rapalogues induces compensatory feedback loops resulting in AKT/ERK activation, which may be abrogated by mTORC2 inhibition. A first-in-human trial evaluating tolerability, pharmacokinetics and pharmacodynamics of the dual TORC1/TORC2 inhibitor OSI-027 was conducted.

**Methods:**

Dose escalation was pursued for three schedules of administration (three consecutive days per week (S1), once a week (S2) and daily dosing (S3)), until dose-limiting toxicities (DLT) were identified. Expansion cohorts with paired tumour biopsies were initiated based on tolerability and pharmacodynamics.

**Results:**

One hundred and twenty eight patients with advanced cancer were enrolled. DLT consisted predominantly of fatigue, renal function disturbances and cardiac events. OSI-027 exposure was dose proportional, with *T*_max_ within 4 h and a half-life of ∼14 h. Expansion cohorts were initiated for S1 and S2, as MTD for S3 was overall considered suboptimal. Target modulation in peripheral blood mononuclear cells were observed from 30 mg, but in tumour biopsies 120 mg QD were needed, which was a non-tolerable dose due to renal toxicity. No RECIST responses were recorded, with stable disease >6 months in six (5%) patients.

**Conclusions:**

OSI-027 inhibits mTORC1/2 in patients with advanced tumour s in a dose-dependent manner but doses above the tolerable levels in S1 and S3 are required for a sustained biological effect in tumour biopsies.

The mammalian target of rapamycin (mTOR) is a well-established target for cancer therapy. mTOR is a 289-KDa serine-threonine kinase that forms part of at least two distinct multiprotein complexes, mTORC1 and mTORC2, both of which regulate distinct branches of the mTOR signalling network (Sabatini, 2006). Altogether, the mTOR complexes are key elements in cell growth, proliferation and survival regulation. Targeting mTOR has demonstrated anti tumour activity in clinical studies; as a result, allosteric inhibitors of mTOR, have been approved for the treatment of renal cell carcinoma, pancreatic neuroendocrine tumours and advanced hormone receptor positive, HER2-negative breast cancer (Hudes *et al*, 2007; Motzer *et al*, 2008; Yao *et al*, 2011; Baselga *et al*, 2012).

Despite the benefit shown in some cancer types, blockade of mTORC1 is not sufficient to completely abrogate signalling of the PI3K/AKT/mTOR pathway (Feldman *et al*, 2009). Indeed, there is evidence that inhibition of mTORC1 by rapalogues results in the release of a negative-feedback loop between S6K and IRS1, leading to phosphorylation of AKT at Thr308 and Ser473, which could be avoided by mTORC2 blockade (Cloughesy *et al*, 2008; Tabernero *et al*, 2008).

Dual mTOR kinase inhibitors are being developed to maximise the pharmacological impact of mTOR blockade, as mTORC2 is insensitive to rapalogues (Jacinto *et al*, 2004; Becker *et al*, 2014; Wilson-Edell *et al*, 2014). OSI-027 (also known as ASP7486) is an orally available small molecule dual mTORC1/mTORC2 ATP-competitive kinase inhibitor, with an IC50 of 22 nM and 65 nM for mTORC1 and mTORC2, respectively. In preclinical studies, OSI-027 demonstrated activity in a broad panel of tumour cell lines, with IC50 values<10 *μ*M, including models resistant to rapalogues. In addition, anti tumour activity was documented in tumour xenograft models. (Bhagwat *et al*, 2011; Falcon *et al*, 2011) Preclinical toxicology studies identified signs of immunosuppression, renal function impairment, glucose metabolism abnormalities as well as potential for cardiac and ocular toxicities at high doses. The maximum tolerated dose (MTD) in toxicology studies was 20 mg kg^−1^ per day in rodents and 2.5 mg kg^−1^ per day in monkeys.

We present here a first-in-man study of the mTORC1/mTORC2-targeting agent OSI-027 in patients with advanced solid malignancies, with the primary objectives of determining a MTD and recommend a dose for phase 2 trials. To optimise tolerability and drug exposure, three schedules of administration were selected for clinical investigation based on the preclinical data.

## Materials and Methods

### Eligibility criteria

Patients with advanced solid tumours refractory to standard therapies were enrolled after providing written informed consent and based on meeting eligibility criteria, including: age ⩾18 years, ECOG-Performance Status 0–2, fasting glucose ⩽7 mmol l^−1^ and left ventricular ejection fraction (LVEF) ⩾60%, by multigated acquisition scan and/or echocardiography; complete eligibility criteria are available in Supplementary Material.

### Trial design overview

Three different schedules of administration were investigated during the dose-escalation phase: once daily for three consecutive days every week (Schedule 1 (S1)), once weekly (Schedule 2 (S2)) and continuous once daily (Schedule 3 (S3)). The primary objective of the study was to determine MTD and recommend a dose and schedule of OSI-027 for phase 2 trials. Secondary objectives included tolerability, safety and pharmacokinetics profile, assessment of pharmacodynamics and preliminary antitumour activity.

Dose escalation was pursued independently for each schedule following a 3+3 design. Escalation was permitted if <33% patients on a dose level experienced dose-limiting toxicities (DLT), defined as any non-haematological adverse event grade ⩾3 (NCI-CTCAE v3) related with the study drug occurring during the first cycle of treatment (21 days), with the exception of non-adequately managed fatigue, nausea, vomiting and/or diarrhoea. In addition, absolute reduction ⩾15% on LVEF was also considered DLT. MTD was defined as the immediately lower dose level to the one where ⩾33% of patients experienced DLT.

Expansion cohorts were planned to provide evidence of pharmacodynamics in tumour tissue as well as to complement the safety data. Dose for expansion cohorts was selected based on MTD determination and the PD data in peripheral blood mononuclear cells (PBMC).

The study was approved by the institutional review boards of all participating centres, and sponsored by Astellas Pharma Global Development (Northbrook, IL, USA), ClinicalTrials.gov Identifier NCT00698243.

### Treatment administered and starting dose

The investigational product was presented in hard gelatine capsules containing 5 or 20 mg of OSI-027 plus microcrystalline cellulose, hydroxypropyl cellulose, croscarmellose sodium, and magnesium stearate as excipients. The starting dose of 10 mg QD three days per week for S1 was selected based on preclinical toxicology studies: 9 mg QD was the equivalent dose for human to 1/6 of the highest non-severely toxic dose in 28-day repeated-dose studies in monkeys. The starting dose for S2 (10 mg QWK) and S3 (5 mg QD) were selected based on preliminary safety data from S1.

### Study procedures

#### Safety assessments

Adverse events and concomitant medications were recorded throughout the trial participation.

Guidelines for dose modifications were implemented; drug administration was halted and/or dose was reduced for creatinine increase grade ⩾2 and/or if absolute values of LVEF fell ⩾15% from baseline. Permanent treatment discontinuation was mandated for LVEF drops ⩾20%.

#### Pharmacokinetic assessments

Blood and urine samples were collected at prespecified time points for 24 h after initial dose and at days 2–3 (S2) and after multiple doses on days 3–4 (S1) and 22–23 (S3) across dose levels. Predose samples were collected on days 8, 15 and 22. Urine samples were collected during cycle 1.

#### Pharmacodynamics studies

Drug-mediated inhibition of the mTORC1 and mTORC2 signalling was evaluated through assessing modulation of phospho-4EBP1 and phospho-PRAS40 in PBMC, as well as phospho-4EBP1 and phospho-S6 in tumour biopsies. Blood aliquots were collected prior to start dosing and at prespecified time points during the first course of treatment. Fresh tumour biopsies were obtained from selected patients during the dose escalation stage and from all patients in the expansion cohorts, at baseline and during cycle 1.

#### Antitumour activity

Assessments by imaging techniques (CT and/or MRI) were performed every 8–12 weeks or earlier if clinically indicated.

### Statistical considerations

Descriptive statistics were used to summarise the safety data, including all patients who received at least one dose of OSI-027. All patients who completed the pharmacokinetic sampling were included in the PK analyses, which used descriptive statistics to summarise AUC0–∞, AUC0–*t*, *C*_max_, *C*_last_, *T*_max_, *T*_last_, %AUC extrapolated, T_1/2_, clearance (*C*l/*F*) and volume of distribution (*V*_z_/*F*). Exploratory analysis were planned to correlate pharmacodynamics and pharmacokinetics. Response to treatment was evaluated according to Response Evaluation Criteria in Solid Tumors (RECIST) (Therasse *et al*, 2000).

## Results

### Patient characteristics

Overall, 128 patients were enrolled in this study between July 2008 and August 2012, with 123 receiving treatment with OSI-027 (56 subjects on S1, 39 on S2 and 28 on S3). The most common primary tumour types among the participants were colorectal (32, 25%), melanoma (13, 10.1%) and renal (11, 8.6%) cancers. The median age of the trial population was 56.5 years; baseline characteristics are presented in Table 1.

### Adverse events and MTD

#### Dose escalation and MTD

**Figure 1:**
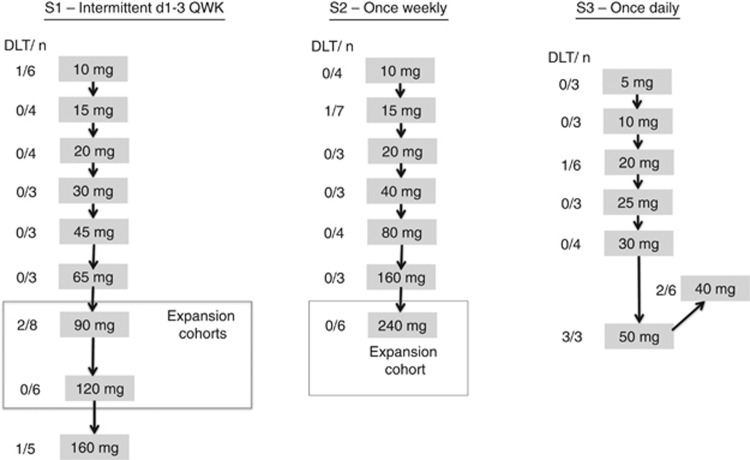
**Schema of the trial design and dose levels explored during the dose escalation phase.** The numbers on the left of each dose level refer to the number of patients treated at dose limiting toxicities in each cohort. Three cohorts were selected for further evaluation in non-randomised expansion cohorts to pursue pharmacodynamics studies in tumour tissue.

Eleven events occurring in 11 patients were judged as dose-limiting toxicities (DLT): grade 3 fatigue (*n*=4 events), grade ⩾2 creatinine rise (2), grade 2 decreased LVEF (1), grade 3 cardiomyopathy (1), grade 3 bone pain (1), grade 3 maculo-papular rash (1) and grade 3 hyperglycaemia (1)) (Figure 1 and Supplementary Table 1).

In schedule 1, nine dose levels (10–160 mg) were explored; one out of six patients treated at the lower dose level (10 mg) experienced a DLT of congestive cardiac failure. At a dose of 90 mg, two out of eight patients had DLT of grade 3 fatigue and grade 3 bone pain. Dose levels of 120 mg and 160 mg were explored; one event of grade 3 hyperglycaemia was identified as DLT at 160 mg. After assessment of the overall toxicity data in the escalation phase of S1, 120 mg was selected as the highest potentially feasible dose and two expansion cohorts were opened, for further evaluation of 90 and 120 mg.

Seven dose levels from 10 to 240 mg QWK were tested in schedule 2. Dose escalation was discontinued before a formal MTD was reached based on the large number of tablets needed. Fifteen additional patients were treated with 240 mg QWK in an expansion cohort.

At 50 mg QD (S3), all three patients treated experienced DLT (grade 3 fatigue, grade 3 urticaria and grade 3 cardiomyopathy). A lower dose level of 40 mg QD was explored, observing grade 2 creatinine elevation in two out of six patients recruited. Therefore, 30 mg QD was considered the MTD for S3.

#### Tolerability profile

Overall, 10 patients (8.1%) discontinued drug permanently due to drug-related adverse events, mostly in S3, and 21 (17%) patients experienced a grade ⩾3 drug-related event. Fatigue and gastrointestinal toxicities (nausea, anorexia and vomiting) were the commonest adverse events and were distributed evenly among the different drug schedules. Also, fatigue and diarrhoea were the more common grade ⩾3 AE (Tables 2A and B)

Disturbances of the renal function were common: creatinine rise (defined as increase of at least one grade in CTCAE v3) was observed in 32 patients (26%) primarily in S3 (29%) and in S1 (32%, most of these in the expansion cohort at 120 mg). (Supplementary Table 2) In contrast, creatinine rise was less common with weekly administration (15%). Renal impairment on treatment was not associated with baseline creatinine levels of the patient, neither with the use of non-steroidal anti-inflammatory drugs nor other baseline concomitant medications. Most of the creatinine elevations were grade 1, appeared during the first course of treatment and were reversible, but one case of grade 4 renal failure was observed in a 53-year-old man with metastatic transitional cell carcinoma of the bladder after 40 days of treatment (S3–40 mg QD). A CT scan demonstrated disease progression and the patient deteriorated rapidly, deceasing 1 week later. Postmortem examination demonstrated tumour infiltration of glomeruli and tumour embolisms in lung, liver and renal capillary vessels.

Cardiac function was closely monitored during the study. A 19-year-old female in the S1–10 mg cohort previously treated with doxorubicin for a metastatic osteosarcoma experienced congestive heart failure after 3 weeks of treatment; her LVEF dropped from 60 to 48% by echocardiogram. Of the 56 patients enrolled in S1, 21 (38%) presented a drop in the LVEF after 3 weeks of treatment, but only in one of these cases the decrease was >15 percentage points. The incidence of LVEF drop was similar among the S1 expansion cohorts (6 out of 14 at 90 mg; 5 out of 14 at 120 mg).

A 67-year-old man in S1–90 mg (reduced to 65 mg due to creatinine rise) with liver and peritoneal metastasis from a gastrointestinal stromal tumour (GIST) suffered a grade 4 myocardial infarction on day 43 of treatment. Last, one patient enrolled in the S3–50 mg cohort developed a reversible stress cardiomyopathy (Takotsubo cardiomyopathy) (Dote *et al*, 1991), which was considered a DLT. On day 15 of treatment, the patient reported retrosternal pain and dyspnoea; the echocardiogram detected an area of anterioapical akinesia with basal hypercontraction and apical ballooning, (LVEF 40%, compared with 65% at baseline); OSI-027 was discontinued and the patient underwent coronary catheterisation, revealing stenosis of the ramus intermedius coronary artery (80%), right coronary artery (25%), and left anterior descending coronary artery (30%). Treatment included antiagregants, bisoprolol, spironolactone, ramipril and atorvastatin. A follow-up echocardiogram 5 days later showed normalisation of the LVEF (68%) and resolution of the apical ballooning.

Drug-related grade 3 hyperglycaemia was only observed in one individual receiving 160 mg QD (S1); cutaneous toxicities, mainly grade 1–2 macular or maculo-papular rash, occurred in 31 (25%) patients.

A total of eight individuals died during study participation (including the post-treatment 30 days follow-up). In seven out of eight cases, the deaths were judged as related to progression of the underlying cancer. The other case was a patient in S3 (40 mg) who died of a post-surgical complication after discontinuing drug due to disease progression.

### Pharmacokinetics of OSI-027

**Figure 2:**
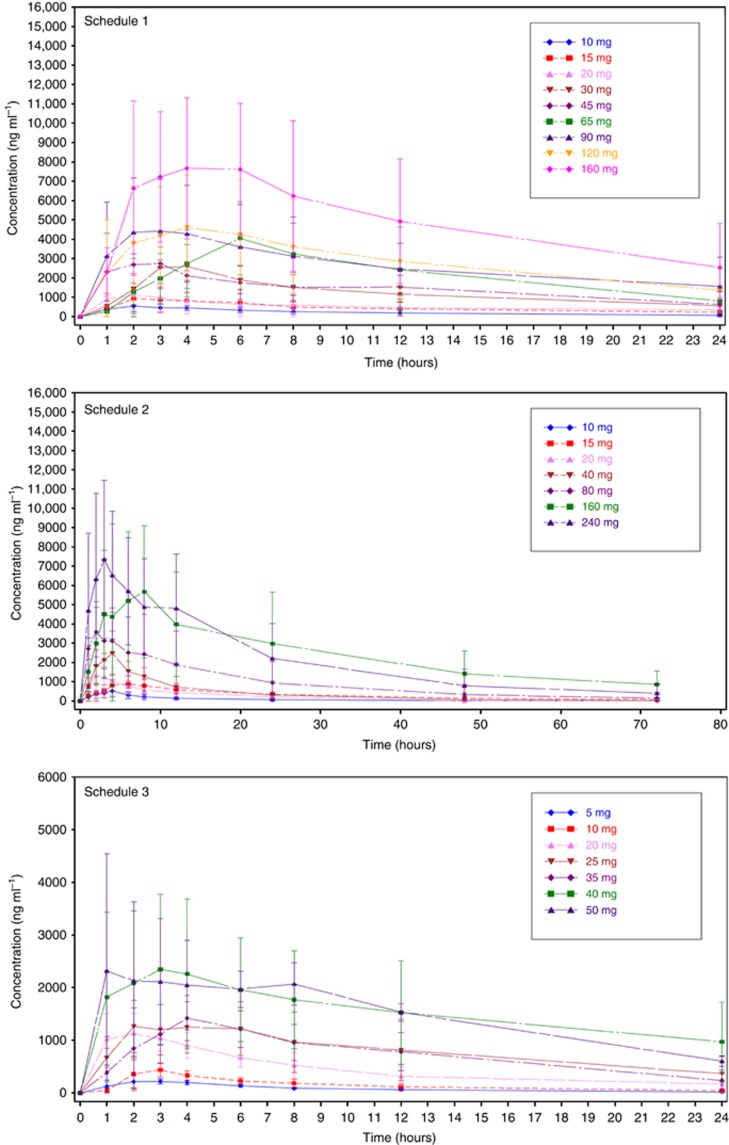
**Mean (and s.d.) concentration in plasma of OSI-027 after first dose until next dose in each of the three schedules of administration.**

All 123 patients dosed with OSI-027 in this study were included in the PK analysis. The pharmacokinetic profile of OSI-027 is summarised in Figure 2 and Supplementary Table 3. OSI-027 was absorbed rapidly, achieving peak plasma concentrations within 4 h. The *T*_max_ was similar across dose groups, suggesting that the rate of absorption of OSI-027 was independent of dose. The estimate of half-life was approximately 14 h (range 8–25 h) with minimal accumulation observed after once daily dosing. Overall, the exposure of OSI-027 was dose proportional and the range of urinary excretion of unchanged OSI-027 was 1–7%.

### Pharmacodynamics in PBMC and tumour tissue

Drug-mediated inhibition of mTORC1 and mTORC2 signalling by OSI-027 was assessed by measuring inhibition of their effectors p4EBP1 (Thr37/46) and pPRAS40 (Thr246), respectively, in PBMC. In addition, paired tumour biopsies at screening and during first month of therapy were used to study target modulation.

**Figure 3:**
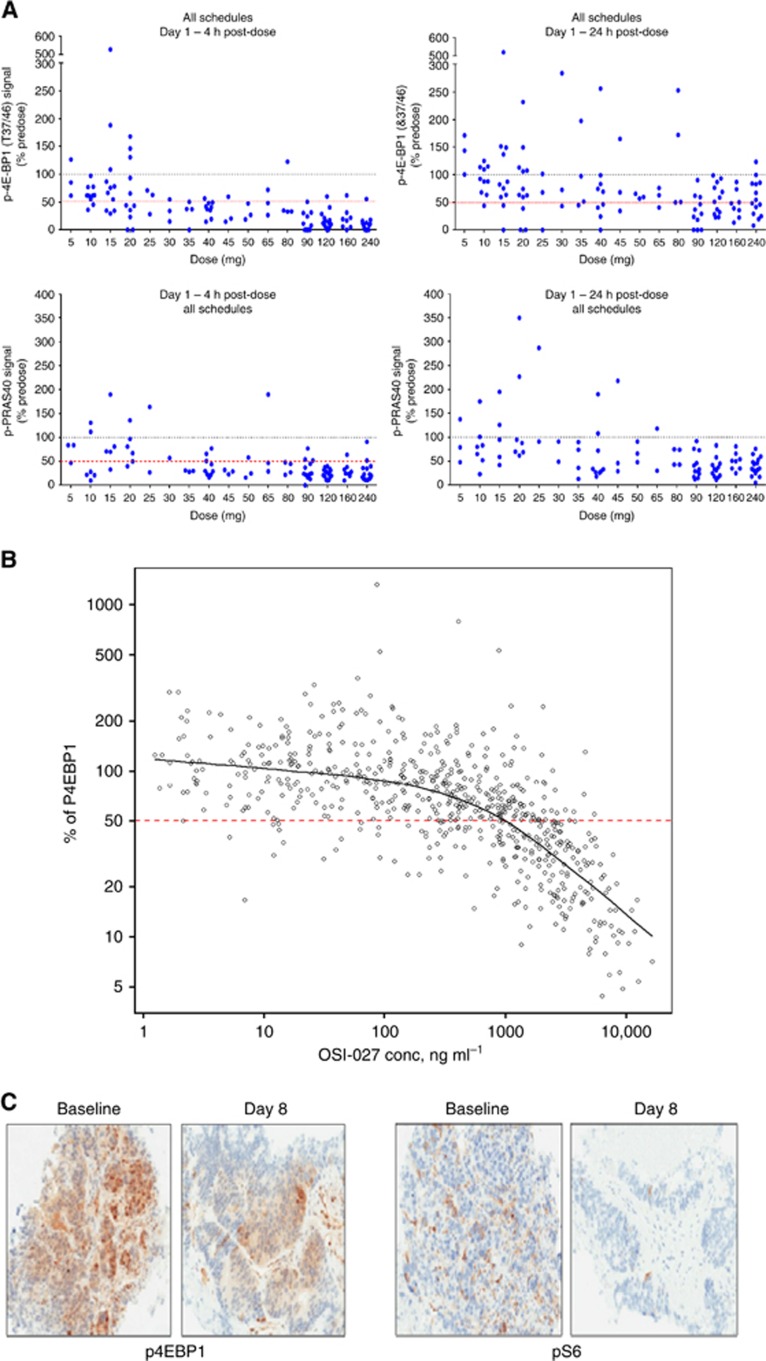
**Pharmacodynamics of OSI-027 and target modulation.** (**A**) Inhibition of pEBP1 (mTORC1 effector, upper panels) and pPRAS40 (mTORC2 effector, lower panels) in PBMC at 4 and 24 h after administration of OSI-027 (all schedules). (**B**) Correlation between plasma concentrations of OSI-027 and reduction of p4EBP1 in PBMC for all patients and all time points. (**C**) Expression of p4EBP1 (left panels) and pS6 (right) by IHC in tumour biopsies; significantly reduced expression is seen in samples taken after 1 week of treatment with 120 mg QD 3 days per week of OSI-027 (S1, pictures on the right) compared with the paired baseline sample of the same patient (pictures on the left).

Inhibition of p4EBP1 and pPRAS40 in PBMC by OSI-027 was dose dependent. Over 50% inhibition of p4EBP1 and pPRAS40 4 h after dosing was almost universal at doses over 30 mg. However, only with doses over 90 mg the effect was maintained over 24 h. When assessing the intermittent schedule (S1), a dose of 90 mg maintained inhibition >50% of p4EBP1 and pPRAS40 during the 3 days on treatment, but after the 4-day break, both markers generally returned to baseline levels. Notably, the highest dose tested (160 mg) was able to maintain >50% target inhibition during the 4-days off treatment in most patients. The once-a-week administration of OSI-027 (S2) induced the maximum inhibition of p4EBP1 and pPRAS40 but the effect was not sustained through next dose. On an average, there was a direct correlation between plasma concentrations of OSI-027 and the inhibition of p4EBP1 in PBMC (Figure 3).

In 21 cases, pre- and postdose paired tumour biopsies were amenable for PD studies. Average reduction in p4EBP1 immunohistochemistry (IHC) staining after drug exposure was 22.3% for 90 mg—S1, 49.5% for 120 mg—S1 and 67.6% for 240 mg—S2, as determined by quantification of digital images and independent confirmation by a pathologist. Similarly, assessment of pS6 IHC showed that although 90 mg only mildly reduced pS6 expression (average 28.6%), 120 mg of OSI-027 achieved stronger target modulation in tumour tissue, (average 75.3% reduction in pS6).

### Preliminary antitumour activity

No complete or partial responses were observed. Thirty-eight (29.7%) patients had stable disease at the first follow-up radiological assessment (8–12 weeks; Supplementary Figure 1). Of these, six patients remained free of progression for ⩾24 weeks, including three patients who remained on therapy for 10–12 months: (1) a woman with metastatic GIST (S1–120 mg) who remained on therapy for 45 weeks with an overall 20% reduction in the sum of her target lesions diameters; (2) a patient with metastatic colorectal cancer (S3–10 mg) with a 18% reduction in the size of the lesions who was on treatment for 49 weeks; and (3) a young patient with metastatic uveal melanoma who was free of progression for 48 weeks (S3–35 mg).

## Discussion

This report presents results from a multicentre, first-in-man clinical trial of OSI-027, a selective mTORC1/mTORC2 inhibitor, designed to overcome the biological limitations of isolated mTORC1 blockade with rapalogues.

Three different schedules of administration were investigated. Although the potentially effective dose of 120 mg QD 3 consecutive days per week (S1) did not result in DLT during the first cycle of treatment, overall it was considered non tolerable as over half of the patients necessitated a dose reduction, mainly due to creatinine elevation. For S2 (once a week schedule), dose escalation was interrupted at 240 mg QWK due to the high number of capsules required. For the continuous daily dosing (S3), renal toxicity was dose limiting above 30 mg QD.

Absorption of OSI-027 was rapid (Tmax within 4 h across dose levels). A terminal half-life of 14 h was estimated based on the weekly dose schedule, with a range of half-life for other groups of 8–25 h. Drug exposure was dose-proportional and there was minimal accumulation after continuous dosing.

Two dose levels, 90 mg and 120 mg, on S1 and the highest administered dose in S2 (240 mg) were selected for further evaluation of pharmacodynamics in non-randomised expansion cohorts. Data from paired tumour biopsies provided evidence of target inhibition at doses of 120 mg; although target modulation in PBMC was observed at doses beyond 30 mg, target inhibition in tumour was suboptimal with 90 mg of OSI-027, suggesting that distribution of active drug to solid tissues was limited relative to circulating blood levels.

The S2 schedule led to the greatest level of target engagement but this was not sustained for the whole weekly treatment interval. While this would preclude single agent efficacy it may be feasible for combination strategies, for example with weekly chemotherapy; acknowledging the risk of increasing renal toxicity.

The tolerability profile of the compound was distinct from the classical effects of rapalogues, as rash, hyperglycaemia or mucositis were infrequent and mild with OSI-027 (Sonis *et al*, 2010; De Masson *et al*, 2011). Liver function disturbances, which have been reported as dose limiting for other mTORC1/mTORC2 inhibitors were not common (Naing *et al*, 2012). In contrast, renal function disturbances represented clearly a major hurdle in this study. The risk of renal impairment due to affection of glomerular permeability or acute tubular necrosis with mTOR inhibition have been described; the exact mechanism has not been fully elucidated but may be related to the role of mTOR signalling in renal regeneration after acute damage (Cinà *et al*, 2012; Lieberthal and Levine 2012). Prior similar compounds tested in clinical trials have not identified renal toxicity as dose-limiting toxicity (Naing *et al*, 2012; Izzedine *et al*, 2013; Basu *et al*, 2015); still, two other cases of acute renal impairment in patients receiving a dual mTORC1/mTORC2 inhibitor were reported following the same pattern observed with OSI-207: acute onset within few days of therapy and rapid normalisation of kidney function after drug discontinuation (Izzedine *et al*, 2013). In this study with OSI-027, intermittent administration of the study drug partially overcame this problem, although eventually creatinine rises almost invariably appeared at higher doses. In this trial, renal toxicity did not seem to be secondary to dehydration or mucositis but a primary drug-related event. Cardiac events were the other major concern in the study; although the frequency was low, three patients experienced severe events. One patient previously treated with doxorubicin developed a Takotsubo cardiomyopathy, a reversible transient systolic dysfunction of unclear pathogenesis, previously related to non-specific forms of stress, including chemotherapies (Dote *et al*, 1991; Qasem *et al*, 2014).

The design of this first-in-man study attempted to optimise the integration of the safety data, pharmacokinetics and pharmacodynamics. The concurrent assessment of various administration schedules, selected based on preclinical data, and the integration of PK/PD correlations is an increasing feature of phase I trial design for targeted agents, aiming to optimise drug delivery and inform subsequent drug combination studies (Molina *et al*, 2011). Moreover, acquisition of paired tumour biopsies permitted the correlation of target modulation in tumour and PBMC: while PBMC studies provided valuable proof-of-mechanism for OSI-027, they did not precisely predict the degree of target engagement in tumour tissue.

In summary, OSI-027 inhibited mTORC1/mTORC2 in a dose-dependent manner but the doses necessary for significant target engagement in tumour were above the tolerable doses in this clinical trial for either continuous daily or three times a week dosing. Further studies of this compound would be necessary to establish any role for once weekly dosing as a potential component of combination strategies, although a revised formulation might be required. However, these would need to be approached cautiously because of the potential for major renal toxicity. On the basis of the PD and tolerability data from this study, further clinical development of the compound as a single agent has been discontinued.

## Figures and Tables

**Table 1:** Baseline characteristics of the trial population

	**n**	**%**
**Gender**		
Male	60	46.9%
Female	68	53.1%
**Age**		
Median (range)	56.5 yo (19–77)	
**ECOG PS baseline**		
0	40	31.3%
1	80	62.5%
2	8	6.3%
**Prior therapies**		
Chemotherapy	126	98.4%
Radiotherapy	57	44.5%
Surgery	111	86.7%
Other	21	16.4%
**Number prior therapies for metastatic/advanced disease**		
Median (range)	2 (1–11)	
1–2	60	46.9%
3–4	40	31.3%
⩾5	28	21.9%
**Primary tumour type**		
Colorectal	32	25.0%
Melanoma	13	10.1%
Renal	11	8.6%
Sarcoma	7	5.5%
Cervix	6	4.7%
GIST	6	4.7%
Ovarian	5	3.9%
Non-small cell lung	3	2.3%
Others	45	35.2%
**Schedule of administration (*n*=123)**		
S1: QD, days 1–3 QW	56	45.5%
S2: QWK	39	31.7%
S3: QD continuously	28	22.8%

Abbreviations: QD=once daily from latin quaque die; QWK=once weekly.

**Table 2A:** Treatment emergent adverse events reported in at least 10% of the trial population.

	**Number of patients, n (%)**			
	**S1 (** ***n*** **=56)**	**S2 (** ***n*** **=39)**	**S3 (** ***n*** **=28)**	**Total (** ***n*** **=123)**
**Gastrointestinal disorders**				
Nausea	38 (67.9)	22 (56.4)	14 (50)	74 (60.2)
Vomiting	24 (42.9)	8 (20.5)	9 (32.1)	41 (33.3)
Constipation	18 (32.1)	6 (15.4)	7 (25)	31 (25.2)
Diarrhoea	13 (23.2)	8 (20.5)	7 (25)	28 (22.8)
Abdominal pain	8 (14.3)	7 (17.9)	6 (21.4)	21 (17.1)
**General disorders**				
Fatigue	41 (73.2)	24 (61.5)	15 (53.6)	80 (65.0)
Anorexia	28 (50.0)	12 (30.8)	10 (35.7)	50 (40.7)
Weight decreased	10 (17.9)	1 (2.6)	6 (21.4)	17 (13.8)
Headache	10 (17.9)	3 (7.7)	5 (17.9)	18 (14.6)
**Laboratory findings**				
Blood creatinine increased	19 (33.9)	8 (20.5)	8 (28.6)	35 (28.5)
Hypokalaemia	7 (12.5)	1 (2.6)	7 (25.0)	15 (12.2)
Anaemia	10 (17.9)	5 (12.8)	2 (7.1)	17 (13.8)
**Musculoskeletal and connective tissue**				
Back pain	8 (14.3)	7 (17.9)	3 (10.7)	18 (14.6)
**Respiratory and thoracic**				
Dyspnoea	11 (19.6)	5 (12.8)	5 (17.9)	21 (17.1)
Cough	8 (14.3)	5 (12.8)	5 (17.9)	18 (14.6)

**Table 2B:** List of grade ⩾3 drug-related adverse events, as per investigator assessment

	**Number of patients, n (%)**			
	**S1 (** ***n*** **=56)**	**S2 (** ***n*** **=39)**	**S3 (** ***n*** **=28)**	**Total (** ***n*** **=123)**
Patients with any Grade ⩾3 drug-related events	8 (14.3)	5 (12.8)	8 (28.6)	21 (17.1)
Fatigue	4 (7.1)	1 (2.6)	2 (7.1)	6 (4.9)
Diarrhoea	1 (1.8)	1 (2.6)	1 (3.6)	3 (2.4)
Blood CK increased	0	2 (5.1)	0	2 (1.6)
Hypophosphatemia	2 (3.6)	0	0	2 (1.6)
Lipase increased	1 (1.8)	0	1 (3.6)	2 (1.6)
Blood amylase increased	1 (1.8)	0	0	1 (0.8)
Bone pain	1 (1.8)	0	0	1 (0.8)
Maculo-papular erythematous rash	1 (1.8)	0	0	1 (0.8)
Hyperglycaemia	1 (1.8)	0	0	1 (0.8)
Myocardial infarction	1 (1.8)	0	0	1 (0.8)
Nausea	0	0	1 (3.6)	1 (0.8)
Pneumonia	0	1 (2.6)	0	1 (0.8)
Renal failure	0	0	1 (3.6)	1 (0.8)
Stress cardiomyopathy	0	0	1 (3.6)	1 (0.8)
Urticaria	0	0	1 (3.6)	1 (0.8)
Vomiting	0	0	1 (3.6)	1 (0.8)

Abbreviation: CK=creatine kinase.
