# Supplementary material for: A first in man, dose-finding study of the mTORC1/mTORC2 inhibitor OSI-027 in patients with advanced solid malignancies
Source: Br J Cancer. 2016 Mar 22;114(8):889–96. doi: 10.1038/bjc.2016.59 (PMC4984800; doi:10.1038/bjc.2016.59)

**SUPPLEMENTARY APPENDIX**

**Supplement to:** Mateo, Olmos, Dumez et al. A first in man, dose-finding study of the mTORC1/mTORC2 inhibitor OSI-027 in patients with advanced solid malignancies.

**Table of contents:**

- Eligibility criteria
- Supplementary Tables 1 to 3
- Supplementary Figure 1

**INCLUSION CRITERIA**

A patient was eligible for enrolment into the study if all of the following criteria were met:

1. Histologically or cytologically documented malignancy (solid tumor or lymphoma) that was now advanced and/or metastatic and refractory to established forms of therapy or for which no effective therapy existed. Patients who had refused available standard therapies or whose disease was potentially sensitive to inhibitors of the mTOR pathway were also deemed eligible.

2. Age 18 years.

3. Eastern Cooperative Oncology Group (ECOG) performance status ≤ 2.

4. Predicted life expectancy of at least 3 months.

5. Patients may have had prior therapy, providing the following conditions were met:

a. Chemotherapy: A minimum of 3 weeks (4 weeks for carboplatin or investigational anticancer agents and 6 weeks for nitrosoureas and mitomycin C) must have elapsed between the end of treatment and registration into this study. Prior tyrosine kinase and/or mTOR inhibitor therapy was permitted, with the minimum 3-week interval as above. Patients must have recovered from any treatment-related toxicities (except for alopecia, and Grade 1 neurotoxicity) prior to registration. Patients who experienced fatigue and/or skin changes (including nail changes) with prior therapy should have recovered to Grade 1, or baseline severity, prior to enrolling in this study.

b. Hormonal therapy: Patients may have had prior anticancer hormonal therapy provided it was discontinued prior to registration into the study. However, patients with prostate cancer with evidence of progressive disease may have continued on therapy that produced medical castration (e.g., goserelin or leuprorelin), provided this therapy was commenced at least 3 months earlier.

c. Radiation: Patients may have had prior radiation therapy provided they had recovered from the acute, toxic effects of radiotherapy prior to registration. A minimum of 21 days must have elapsed between the end of radiotherapy and registration into the study unless the radiation affected less than 25% of bone marrow.

d. Surgery: Previous surgery was permitted provided that wound healing had occurred prior to registration.

6. Adequate hematopoietic, hepatic, and renal function, defined as follows:

 Neutrophil count ≥ 1.5 times 10^9^/L and platelet count ≥ 100 times 10^9^/L;

 Bilirubin ≤ 1.5 times upper limit of normal (ULN);

 Aspartate aminotransferase (AST) and/or alanine aminotransferase (ALT) ≤ 2.5 times ULN or ≤ 5 times ULN if the patient had documented liver metastases; and

 Serum creatinine within the normal range for the institution.

7. Fasting glucose ≤ 125 mg/dL (7 mmol/L) at baseline.

8. Left ventricular ejection fraction (LVEF) by electrocardiogram (echocardiogram

[ECHO]) or multiple gated acquisition scan (MUGA) ≥ 60% (if MUGA scan utilized for eligibility, ECHO must have been performed at baseline for comparison to on-treatment ECHOs).

9. Female patient must have been either

 Of non-childbearing potential:

- postmenopausal (defined as at least 1 year without any menses) prior to screening, or
- documented surgically sterile or status post hysterectomy (at least 1 month prior to screening)

 Or, if of childbearing potential:

- must have had a negative urine pregnancy test at screening, and
- must have used 2 forms of birth control (at least one of which was a barrier method) starting at screening and throughout the study period and for 28 days (or 5 half-lives of the study drug, whichever was longer) after the final study drug administration. Highly effective contraception was defined as established use of oral, injected or implanted hormonal methods of contraception; placement of an intrauterine device or intrauterine system; or barrier methods of contraception (condom or occlusive cap [diaphragm or cervical/vault caps] with spermicidal foam/gel/film/cream/suppository).

10. Female patient must not have been breastfeeding at screening or during the study period and for 28 days or 5 half-lives of the study drug, whichever was longer, after final study drug administration.

11. Female patient must not have donated ova starting at screening and throughout the study period and for 28 days or 5 half-lives of the study drug, whichever was longer, after final study drug administration.

12. Male patient and their female spouse/partners who were of childbearing potential must have been using highly effective contraception consisting of 2 forms of birth control (one of which must have been a barrier method) starting at screening and continuing throughout the study period and for 28 days or 5 half-lives of the study drug, whichever was longer, after final study drug administration.

13. IRB/IEC-approved written informed consent and privacy language as per national regulations (e.g., Health Insurance Portability and Accountability Act [HIPAA] Authorization for United States sites) was obtained from the patient or legally authorized representative prior to any study-related procedures (including withdrawal of prohibited medication, if applicable).

14. Patient agreed not to participate in another interventional study while on treatment.

**EXCLUSION CRITERIA**

A patient was excluded from the study if any of the following criteria were met:

1. History of significant cardiac disease unless the disease was well controlled. Significant cardiac disease included second-/third-degree heart block; significant ischemic heart disease; QTc interval > 450 msec at baseline; poorly controlled hypertension; right or left bundle branch block; or congestive heart failure of New York Heart Association (NYHA) class 2 or worse (slight limitation of physical activity; comfortable at rest, but ordinary physical activity results in fatigue, palpitation, or dyspnoea).
2. Discontinuation from prior therapy (e.g., anthracycline or trastuzumab) due to cardiac toxicity.
3. Active or uncontrolled infections.
4. Serious illnesses or medical conditions that could have interfered with the patient’s on going participation in the study.
5. History of any psychiatric condition that might have impaired the patient’s ability to understand or to comply with the requirements of the study or to provide informed consent.
6. Documented history of diabetes mellitus.
7. Pregnant or breastfeeding females.
8. Symptomatic brain metastases that were not stable, required steroids, or required radiation within the last 28 days.
9. Chronic systemic steroid use for a cancer-related condition. Patients taking chronic systemic steroids for a non cancer-related condition were allowed on the study as long as the schedule was stable.
10. History of allergic reactions attributed to compounds of similar chemical or biologic composition to the study drug.
11. Patients with cataract who were expected to undergo cataract surgery within 6 months of registration.
12. Use of drugs that had a risk of causing QT interval prolongation within 14 days prior to day 1 dosing.
13. Clinically significant electrolyte imbalances (due to the increased risk of arrhythmia if QTc was prolonged). Thus, serum potassium, magnesium and calcium were monitored and corrected as necessary, both prior to enrolment and while on the study.
14. Participation in any interventional clinical study or treatment with any investigational drugs within 30 days or 5 half-lives, whichever was longer, prior to the initiation of screening.

**Supplementary Table 1.** Dose limiting toxicities observed during the dose-escalation phase.

| Schedule | Dose | Days of treatment | Dose limiting toxicity |
| --- | --- | --- | --- |
| S1 | 10 mg | 22 | Decreased LVEF (G2) |
| S1 | 90 mg | 19 | Asthenia / Fatigue (G3) |
| S1 | 90 mg | 14 | Bone pain (G3) |
| S1 | 160 mg | 11 | Hyperglycaemia (G3) |
| S2 | 15 mg | 19 | Fatigue (G3) |
| S3 | 20 mg | 13 | Fatigue (G3) |
| S3 | 40 mg | 12 | Elevated serum creatinine (G2) |
| S3 | 40 mg | 19 | Elevated serum creatinine (G2) |
| S3 | 50 mg | 11 | Fatigue (G3) |
| S3 | 50 mg | 15 | Stress-induced cardiomyopathy (G3) |
| S3 | 50 mg | 21 | Urticaria (G3) |

**Supplementary Table 2.** Summary of changes in renal function during treatment with OSI-027 (across all dose levels and schedules)

|  | *Maximum creatinine on treatment (CTCAE grade)* | | | | | |
| --- | --- | --- | --- | --- | --- | --- |
| Baseline creatinine (CTCAE grade) | *0* | *1* | *2* | *3* | *4* |  |
| 0 | 53 (46.1%) | 50 (43.5%) | 11 (9.6%) | 1 (0.9%) | 0 | 115 |
| 1 | 0 | 4 (50%) | 4 (50%) | 0 | 0 | 8 |
| Total | 53 (43.1%) | 54 (43.9%) | 15 (12.2%) | 1 (0.8%) | 0 | 123 |

**Supplementary Table 3.** Pharmacokinetic profile of OSI-027; all numbers represent median values for each variable, with range in brackets.

| **Schedule 1** | **10 mg** | **15 mg** | **20 mg** | **30 mg** | **45 mg** | **65 mg** | **90 mg** | **120 mg** | **160 mg** |
| --- | --- | --- | --- | --- | --- | --- | --- | --- | --- |
| **Day 1** | *N=6* | *N=4* | *N=4* | *N=3* | *N=3* | *N=3* | *N=14* | *N=14* | *N=5* |
| AUC_inf_  (hr.ng/mL) | 5129  (2556-13739) | 8462  (3953-74585) | 5776  (3433-70625) | 45129  (15013-57926) | 31147  (22561-92378) | 50040  (29070-71011) | 90275  (21439-427286) | 87627  (17889-201833) | 131223  (26804-387469) |
| C_max_  (ng/mL) | 646  (363-1140) | 1171  (418-1760) | 804  (647-2890) | 3620  (1500-3660) | 2770  (2760-4490) | 4750  (3270-5490) | 5440  (2284-11300) | 5594  (1414-9599) | 9329  (3272-13774) |
| T_1/2_  (hr) | 5.98  (4.13-12.5) | 7.12  (6.14-50.3) | 5.09  (3.95-26.3) | 11.3  (4.74-16.2) | 7.65  (6.99-22.2) | 7.01  (6.05-7.96) | 12.1  (5.82-32.7) | 10.2  (4.19-26.1) | 10.3  (5.52-18.9) |
| **Day 3** | *N=6* | *N=4* | *N=4* | *N=3* | *N=3* | *N=3* | *N=14* | *N=13* | *N=5* |
| AUC_24hr_  (hr.ng/mL) | 4859  (2775-12591) | 9212  (4304-45594) | 5848  (3440-51834) | 37756  (15019-55001) | 35150  (22328-80913) | 64427  (33206-80259) | 69565  (28271-200596) | 54477  (17985-202476) | 105663  (33245-264755) |
| C_max_  (ng/mL) | 602  (449-954) | 1274  (490-2940) | 1349  (830-4050) | 2120  (1880-4500) | 3850  (3710-4920) | 5550  (3330-7260) | 7014  (3920-14000) | 5449  (1633-12671) | 11007  (4014-18702) |
| T_1/2_  (hr) | 7.74  (4.22-10.6) | 7.69  (6.97-27.0) | 5.97  (5.02-21.9) | 10.0  (5.76-14.3) | 9.14  (8.06-16.4) | 8.46  (7.65-9.27) | 12.0  (7.97-23.7) | 12.8  (4.68-33.3) | 11.9  (4.25-14.4) |
| **Schedule 2** | **10 mg**  **N=4** | **15 mg**  **N=7** | **20 mg**  **N=3** | **40 mg**  **N=3** | **80 mg**  **N=4** | **160 mg**  **N=3** | **240mg**  **N=15** |  | |
| AUC_inf_ (hr.ng/mL) | 5531  (1619-8900) | 25892  (11794-36702) | 9843  (3560-38678) | 28592  (18949-34332) | 52639  (10384-150342) | 267414  (22103-318275) | 125278  (33760-388068) |
| C_max_  (ng/mL) | 705  (432-778) | 894  (478-1670) | 1240  (876-2770) | 2510  (2360-3760) | 3890  (3150-5390) | 7620  (1710-7780) | 8780  (4908-14010) |
| T_1/2_  (hr) | 12.3  (6.95-17.6) | 19.3  (9.49-32.0) | 7.65  (7.56-18.0) | 12.4  (11.4-13.7) | 9.22  (7.47-18.5) | 24.7  (17.0-32.3) | 13.8  (7.39-30.8) |
| **Schedule 3** | **5 mg** | **10 mg** | **20 mg** | **25 mg** | **35 mg** | **40mg** | **50 mg** |
| **Day 1** | *N=3* | *N=3* | *N=6* | *N=3* | *N=4* | *N=6* | *N=3* |
| AUC_inf_  (hr.ng/mL) | 2143  (1535-2740) | 4303  (2570-4881) | 9457  (8564-39407) | 25106  (7556-53093) | 20205  (7524-28796) | 77137  (10140-268074) | 46250  (41232-51268) |
| C_max_  (ng/mL) | 236  (184-321) | 547  (287-723) | 1300  (952-1580) | 1640  (831-1800) | 1590  (924-1780) | 3140  (1000-4420) | 3040  (2530-4590) |
| T_1/2_  (hr) | 7.89  (4.97-8.33) | 7.24  (5.07-9.14) | 7.03  (5.92-23.1) | 11.1  (5.51-19.3) | 7.24  (3.88-9.68) | 17.8  (5.67-67.3) | 10.3  (9.27-11.3) |
| **Day 22** | *N=3* | *N=3* | *N=5* | *N=3* | *N=4* | *N=4* | *N=1* |
| AUC_24hr_  (hr.ng/mL) | 2044  (2013-2755) | 3717  (3249-4490) | 13704  (11176-21020) | 15169  (10719-35141) | 31219  (7733-45364) | 50132  (10928-104286) | 17246 |
| C_max_  (ng/mL) | 266  (207-276) | 457  (368-513) | 1540  (1310-1800) | 1790  (1210-1800) | 2300  (1010-3780) | 3005  (2440-5350) | 1110 |
| T_1/2_  (hr) | 6.85  (5.68-8.01) | 6.54  (5.68-12.6) | 9.67  (7.93-10.9) | 15.0  (6.84-50.7) | 11.1  (5.70-12.8) | 15.9  (7.75-38.0) | - |

**Supplementary Figure 1**. Preliminary antitumor activity of OSI-027; bar plot representing the time on treatment (weeks) of the ten patients who remained on therapy for > 12 weeks.


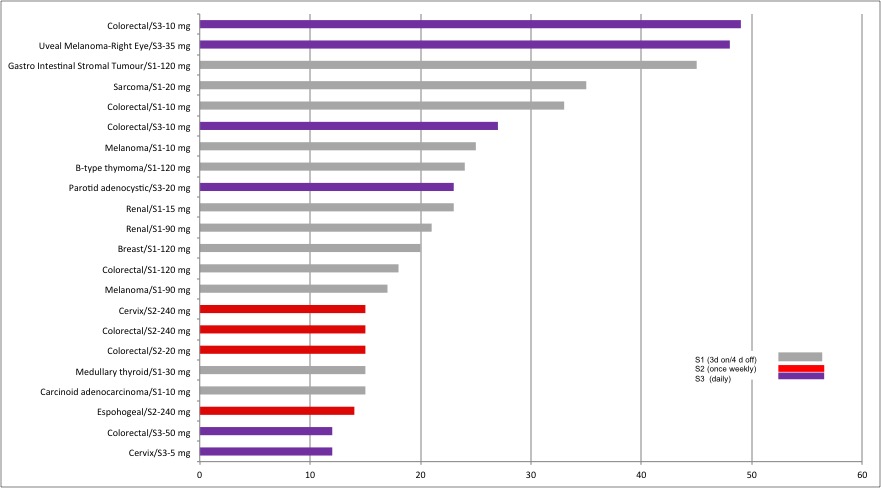

Supplement: Supplementary Information [file bjc201659x1.doc]
